# Head‐up tilt does not enhance prostate tumor perfusion or oxygenation in young rats

**DOI:** 10.14814/phy2.15548

**Published:** 2022-12-23

**Authors:** Olivia N. Kunkel, Taylor A. Rand, Joseph G. Pyle, Dryden R. Baumfalk, Andrew G. Horn, Alexander B. Opoku‐Acheampong, Carl J. Ade, Timothy I. Musch, Michael W. Ramsey, Michael D. Delp, Bradley J. Behnke

**Affiliations:** ^1^ Department of Kinesiology Kansas State University Manhattan Kansas USA; ^2^ Department of Anatomy and Physiology Kansas State University Manhattan Kansas USA; ^3^ Department of Sport, Exercise, Recreation, and Kinesiology East Tennessee State University Johnson City Tennessee USA; ^4^ Department of Nutrition, Food and Exercise Sciences Florida State University Tallahassee Florida USA; ^5^ Johnson Cancer Research Center Kansas State University Manhattan Kansas USA

## Abstract

Solid tumors contain hypoxic regions that contribute to anticancer therapy resistance. Thus, mitigating tumor hypoxia may enhance the efficacy of radiation therapy which is commonly utilized for patients with prostate cancer. Increasing perfusion pressure in the prostate with head‐up tilt (HUT) may augment prostate tumor perfusion and decrease hypoxia. The purpose of this study was to determine if an increase in the vascular hydrostatic gradient via 70° HUT increases tumor perfusion and decreases tumor hypoxia in a preclinical orthotopic model of prostate cancer. Male Copenhagen rats (*n* = 17) were orthotopically injected with Dunning R‐3327 (AT‐1) prostate adenocarcinoma cells to induce prostate tumors. After tumors were established, prostate tumor perfusion and hypoxia were measured in rats during level (0°) and 70° HUT positions. To compare the magnitude of the hydrostatic column to that present in humans, ultrasound was used to measure the heart to prostate distance in male human subjects to estimate the prostate vascular hydrostatic pressure with the upright posture. In young rats, no differences were detected in prostate tumor perfusion or prostate tumor hypoxia with 70° HUT versus the level position. However, from the retrospective study, young rats increased prostate vascular resistance to HUT, whereas aged rats lacked this response. Tumor vessels co‐opted from existing functional vasculature in young rats may be sufficient to negate increases in perfusion pressure with HUT seen in aged rats. Additionally, in humans, the estimated hydrostatic column at the level of the prostate is five times greater than that of the rat. Therefore, 70° HUT may elicit increases in prostate/prostate tumor blood flow in humans that is not seen in rats.

## Graphical abstract

Blood flow is largely dependent on perfusion pressure, which can be manipulated by body position. Here, we tested the hypothesis that, with head‐up tilt, the increased hydrostatic pressure in orthotopic prostate tumors would increase tumor blood flow. Despite slightly elevated perfusion pressures, we did not observe significant increases in tumor blood flow or reductions in hypoxia.

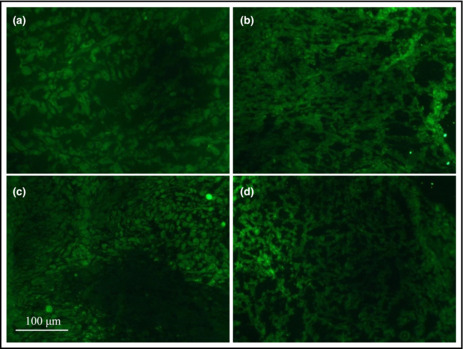

## 1 INTRODUCTION

Although one of the most treatable cancers when diagnosed early, prostate cancer remains the second‐most most diagnosed and second‐leading cause of cancer‐related deaths in men in the United States (Siegel et al., 2018). The hypoxic nature of prostate tumors can present a significant challenge to treatment efficacy, increase metastasis, and serve as a negative prognostic factor for patient survival (Höckel & Vaupel, 2001; Siemann, 2011; Turaka et al., 2012; Vaupel, 2004a, 2004b; Vaupel et al., 2004; Vaupel & Harrison, 2004; Walsh et al., 2014). Patients with hypoxic tumors (i.e., median tumor partial pressure of oxygen (PO_2_) ≤ 10 mmHg) have a significantly lower 5‐year survival rate compared to patients with a median tumor PO_2_ above 10 mmHg (Höckel & Vaupel, 2001; Vaupel, 2004b). Hypoxia in tumors results in several deleterious effects, such as tumor cell migration, increased production of cancer‐associated fibroblasts, and activation of growth factors and transcription factors (Bristow & Hill, 2008; Siemann, 2011), contributing to abnormal angiogenesis (Semenza, 2003; Vaupel, 2004b) and resulting in aberrant, tortuous, heterogenous, and overall immature vasculature (Baish et al., 1996; Baluk et al., 2003; Carmeliet & Jain, 2000; Jain, 1988; Konerding et al., 1999; Vaupel, 2004a; Vaupel & Harrison, 2004; Yancopoulos et al., 2000). This dysfunctional vasculature is a significant contributor to sustained tumor hypoxia and treatment resistance (Li et al., 2000; Siemann, 2011).

One of the primary treatment options for prostate cancer patients is radiation therapy. Although radiation treatment has advanced significantly in recent years, the efficacy of radiation therapy is largely dependent on the presence of oxygen (Thomlinson & Gray, 1955). Thus, in hypoxic tumors, the effectiveness of radiation is significantly diminished due to the lack of available oxygen. Radiation is administered to prostate cancer patients in a supine position, as it ensures targeted administration with minimal damage to surrounding tissue. In the supine position, there are minimal perfusion pressure gradient differences throughout the body due to a near‐constant hydrostatic column (i.e., minimal vertical heart‐to‐tissue distance).

It is well‐known that postural changes creating a hydrostatic column induce redistributions in blood volume due to changes in transmural pressure (Musacchia et al., 1985; Ramsey et al., 2007; Watenpaugh & Hargens, 1996; Wilkerson et al., 1999). In response to the upright posture, tissue below the level of the heart is subjected to a greater transmural pressure, due to increased hydrostatic pressure, but a pronounced local vascular myogenic and/or sympathetically mediated vasoconstriction minimizes blood volume pooling in those tissues (Rowell, 1993). However, preexisting vascular dysfunction limits the ability to offset an increase in vascular hydrostatic pressure with tilt or the upright posture, leading to reductions in blood pressure and potentially orthostatic intolerance (Jansen et al., 1989; Kalbfleisch et al., 1977; Lipsitz, 1985; Minson et al., 1999; Ramsey et al., 2007). In prostate cancer, we have demonstrated a general lack of functional vascular smooth muscle in tumor feed arteries (McCullough et al., 2013, 2014). Further, when prostate tumor feed arteries are subjected to elevated intraluminal pressures, there is a diminished myogenic vasoconstriction versus feed arteries of the healthy prostate (McCullough et al., 2014). Therefore, with the assumption of the upright position and an increased heart‐to‐prostate hydrostatic column, if perfusion pressure is significantly elevated concomitant with tumor arteriolar dysfunction, tumor perfusion may increase and, subsequently, reduce tumor hypoxia. Although there is a smaller absolute difference in heart‐to‐prostate distance for the rat versus human, previous research has shown differences in below‐heart tissue hemodynamics in response to tilt in the rat, including reproductive organs, albeit only with old age (Ramsey et al., 2007). Therefore, we hypothesized that in an orthotopic rat model of prostate cancer, 70° head‐up tilt (HUT) would result in an increase in prostate tumor perfusion and reduction in tumor hypoxia compared to the level position. To establish translational changes in pressure gradients, we measured the heart‐to prostate distance in healthy males to estimate the supine‐to‐upright posture hydrostatic vascular pressure gradient.

## 2 METHODS

### 2.1 Animals

Immunocompetent male Copenhagen rats (6–8 months, *n* = 17; Charles River) were individually housed at 23°C on a 12:12 h light–dark cycle and provided water and standard rat chow ad libitum. All procedures were approved by the Institutional Animal Care and Use Committee at Kansas State University and complied with the National Institutes of Health *Guide for the Care and Use of Laboratory Animals*.

### 2.2 Tumor model

The cell line utilized in this study was the rat‐specific Dunning R‐3327 (AT‐1) prostate adenocarcinoma, which resembles human prostate cancer growth and metastatic potential (Isaacs et al., 1978). AT‐1 cells were cultured in RPMI‐1640 media (GE Healthcare Life Sciences) supplemented with 10% fetal bovine serum (FBS; RMBIO), 2 mM L‐glutamine (Fisher Scientific), 100 mM sodium pyruvate (Thermo Fisher Scientific), 1% penicillin/streptomycin (Thermo Fisher Scientific), and 0.025 mM dexamethasone (Cayman Chemical). Cells were incubated at 37°C with 5% CO_2_ until reaching ~80% confluency. Cells were then counted using a hemocytometer and diluted to 100,000 cells/ml in physiological saline. The solution was then divided into 0.1 ml aliquots to contain 10^4^ AT‐1 cells. Animals were anesthetized with 2%–5% isoflurane/O_2_ balance and a small incision of ~1 cm was made in the abdomen lateral to the midline. The bladder/prostate complex was exposed, and 10^4^ cells were injected into the ventral lobe of the prostate using a sterile 26G syringe. Following injection, the abdominal wall was closed with sterile 4‐0 polyglycolic acid‐coated suture (DemeTECH) and the overlying skin and fascia was closed using sterile 4‐0 nylon monofilament (DemeTECH) and sealed using Vetbond skin adhesive (3 M). Rats were then injected subcutaneously with 0.05 mg/kg buprenorphine (Patterson Veterinary) and 0.5 mg/kg acepromazine (Patterson Veterinary) for analgesia and sedation, respectively, and isoflurane was then withdrawn. All procedures were performed under aseptic conditions and daily postoperative monitoring occurred for 7 days. Tumors were allowed to develop for 6–8 weeks before the experimental protocol.

### 2.3 Blood flow with 70° HUT

Prior to HUT, animals (*n* = 6) were habituated to the tilt apparatus, a Plexiglas canopy universal rat restrainer (Braintree Scientific, Inc.), at 0° tilt for 20 min per day for at least 3 days. At the conclusion of the habituation period, rats were anesthetized with 2%–5% isoflurane/O_2_ balance and a catheter (Silastic, ID 0.6 mm, OD 1.0 mm; Dow Corning) filled with heparinized saline was advanced into the ascending aorta via the right carotid artery, as previously described (Ramsey et al., 2007). This catheter was used for infusion of fluorescent microspheres for tissue blood flow measurements and for the monitoring of mean arterial pressure (MAP). A second catheter (ID 0.36 mm, OD 0.84 mm; Braintree Scientific) was placed in the caudal artery as previously described (Ramsey et al., 2007). The caudal artery catheter was used to obtain a reference blood sample for calculation of tissue blood flows (see below). Both catheters were externalized at the base of the tail and secured. Animals were given 2 h to recover before initiation of the experimental protocol and injected with a low dose of acepromazine (0.2–0.3 mg/kg) to minimize stress and movement within the tilt apparatus.

After this 2 h recovery, animals were placed in the restrainer which was hinged to a tilting support base (i.e., ring stand) in a horizontal standing position (level). The head end of the canopy has a tapered opaque plastic hood to minimize visual disturbances and reduce ocular postural input, which may alter cardiovascular reflex responses (Martel et al., 1998; Wilkerson et al., 1999). After 20 min in the level position, the first microsphere infusion occurred. Thereafter, the tilt position was raised from level to 70° HUT. After 10 min of HUT, the second microsphere infusion occurred, as previous research indicates that 10 min is an appropriate amount of time to reach a cardiovascular steady state in response to HUT (Ramsey et al., 2007). Heart rate and MAP were monitored throughout the experiment and recorded immediately before and after each microsphere infusion. At the end of the experiment, animals were anesthetized with 5% isoflurane/O_2_ balance, the distance between the carotid artery cannula and the prostate tumor was measured, and animals were killed via cardiac excision. The kidneys, soleus muscles, prostate tumor, and viable prostate tissue were excised, weighed, snap frozen in liquid nitrogen, and stored at −80°C for later determination of tissue blood flow as described below.

### 2.4 Determination of blood flow

Fluorescent microspheres (15 ± 0.5 μm diameter; ThermoFisher Scientific) were used for blood flow measurements and vortexed prior to injection. For each injection, a reference blood sample was taken from the caudal artery at a rate of 0.25 ml/min with a withdrawal pump (Harvard Apparatus) 30 s prior to injection of approximately 2.5 × 10^5^ microspheres into the carotid catheter. Warm saline (approximately 0.25 ml) was injected over a 30 s period immediately after microsphere injection to clear the catheter of residual microspheres. Withdrawal of the reference blood sample continued for 20 s after the saline infusion. After killing and tissue dissection, microsphere extraction was performed according to manufacturer's guidelines. Thereafter, sample fluorescence was measured in a spectrophotometer and tissue blood flows were calculated as follows:

$$Q=\left[\left(A_{t}/A_{b}\right)\times \left(s/w\right)\right]\times 100,$$

where *Q* is blood flow (ml/min/100 g), *A*_t_ the individual sample intensity, *A*_b_ reference blood sample intensity, *s* the reference blood sample withdrawal rate (ml/min), and *w* the tissue wet weight (g) (Deveci & Egginton, 1999).

MAP was electronically averaged from pulsatile pressure measurements made via pressure transducer (ADInstruments). Estimated tumor arterial pressure was calculated from the estimated increase in pressure at the level of the tumor based on the average distance between the heart and prostate tumor (see below). Vascular conductance and vascular resistance were calculated as follows:

VC=Q/P,

where VC is vascular conductance (ml/min/100 g/mmHg), Q is blood flow (ml/min/100 g), and P is estimated tumor pressure (mmHg);

VR=P/Q,

where VR is vascular resistance (mmHg/ml/min/100 g), *P* the estimated tumor pressure (mmHg), and *Q* the blood flow (ml/min/100 g).

### 2.5 Tumor hypoxia with 70° HUT

In a second group of animals, an intraperitoneal injection (60 mg/kg) of Hypoxyprobe‐1™ (HP‐1; Hypoxyprobe) was given immediately prior to the experimental protocol and animals were assigned to the level (*n* = 4) or HUT (*n* = 7) position. Multiple injections of Hypoxyprobe‐1™ in the same animal are unable to be distinguished from one another, necessitating the use of separate animals at each tilt position. Animals remained in either the level or HUT position for 45 min. This duration was chosen as it is equal to the half‐life of the HP‐1 compound in plasma, ensuring adequate time for binding in hypoxic tissues while also minimizing interference. After completion of the experimental protocol, animals were anesthetized under 5% isoflurane/O_2_ balance and killed via cardiac excision. The prostate tumor was immediately removed and covered in optimal cutting temperature (OCT) compound (Sakura Finetek) snap frozen in isopentane, and stored at −80°C for later immunohistochemical (IHC) analysis.

To determine the hypoxic cell count of the prostate tumor, IHC analysis was performed according to the methods of Ljungkvist et al. (2005) and Hypoxyprobe, Inc. Using a cryostat, tumors were cut into 4 μm sections and mounted on slides. Two core and two periphery slides, each with three sections, were produced for each tumor. Core sections were sliced consecutively from the center of the tumor, while periphery sections were sliced consecutively from the exterior of the tumor. After fixation, the slides were then rinsed with phosphate buffered saline (PBS) and incubated overnight at 4°C with rabbit anti‐pimonidazole antisera (PAb2627AP diluted 1:20 in PBS containing 0.1% bovine serum albumin and 0.1% Tween‐20). Sections were then incubated for 60 min with FITC conjugated goat anti‐rabbit antibody. Slides were examined under a fluorescent microscope (Zeiss) at 20× magnification with a FITC filter (470–520 nm) and six images were taken from two periphery and two core slides from each tumor. Images were analyzed using ImageJ software (NIH) to calculate an average hypoxic cell count for tumor core and tumor periphery.

### 2.6 Human and rat heart to prostate distance measurements and estimated hydrostatic pressure differences

The heart to prostate distance was measured in both animal and human subjects to determine the magnitude of differences in the hydrostatic pressure gradient from level to HUT positions. In animals subjected to blood flow measurements, the distance between the carotid catheter tip and the prostate was measured. From this measurement, the vertical component of the heart to prostate distance in 70° HUT was calculated as the sin(70°)* measured distance in centimeters. Estimated hydrostatic pressure was calculated from the following equation:

$$P=\left(\sin\left(70\right)\times d\right)\times 0.787$$

where *P* is pressure (mmHg), *d* the distance (cm), and pressure increases by 0.787 mm Hg per cm vertical distance.

The heart to prostate distance of humans was also measured in eight male subjects (all procedures were approved by the Institutional Review Board of Kansas State University). The location of the left atrioventricular valve was determined by echocardiography and the location was marked on the dorsum of each subject. The location of the prostate was determined by palpation of the distal end of the coccyx, and the distance between the two points was measured. Estimated hydrostatic pressure was calculated from the above equation.

### 2.7 Retrospective analysis

To investigate the impact that aging may have on the vascular response of the prostate to HUT, we performed a secondary analysis of age‐related changes in prostate blood flow responses to an identical tilt protocol, originally conducted by Ramsey et al. (2007). Specifically, we determined total prostate vascular resistance in response to 70° HUT in young versus aged animals, which was not previously reported, using the estimated arterial pressure at the level of the prostate (i.e., corrected for changes in the heart‐to‐prostate differences in the vascular hydrostatic pressure gradient) with HUT.

### 2.8 Statistics

For all blood flow data, a two‐tailed paired t‐test was used to determine differences between level and HUT. For hypoxia data, a two‐way ANOVA was used to determine differences in hypoxia between level and HUT and followed by Holm–Sidak post hoc test. A two‐way ANOVA was used to determine differences in tumor core versus periphery in either level or HUT. A Grubbs test was used for detection of outliers. All data are presented as mean ± SE. Significance was set at *p* ≤ 0.05.

## 3 RESULTS

### 3.1 Animal characteristics

Average body weight of animals used in the blood flow portion of the study was 271 ± 9 g with an average tumor weight of 7.1 ± 1.6 g. Average body weight of rats used for hypoxia studies was 300 ± 10 g for the level position and 329 ± 14 g for HUT. There was no difference in body weight between animals used in the level and HUT position (*p* = 0.58; Table 1). For tumor hypoxia measures, separate animals had to be used for level versus HUT, and tumor weight as a percentage of body weight was significantly greater for animals in the level position compared to animals in the HUT position (15 ± 2.9 vs 9.4 ± 0.7 g, respectively; *p* = 0.02; Table 1).

**TABLE 1:** Body weight, tumor weights, and relative tumor weight of animal characteristics.

	Blood flow	Hypoxia	
		0°	70°
*n*	6	4	7
Body weight (g)	271 ± 9	300 ± 10	329 ± 14
Tumor weight (g)	7.1 ± 1.6	15 ± 2.9	9.4 ± 0.7*
Relative tumor weight (% body weight)	2.6 ± 0.6	5.0 ± 1.2	2.9 ± 0.3*

*Note*: Data presented as mean ± SEM. Body weight, tumor weight, and relative tumor weight for animals. Tumor weight and relative tumor weight were significantly different between animals in the level and HUT position (*p* ≤ 0.05). Data are mean ± SE.

* *p* ≤ 0.05 vs hypoxia, 0°.

### 3.2 Blood flow with HUT

#### 3.2.1 Heart rate, MAP, and tissue blood flows

**FIGURE 1:**
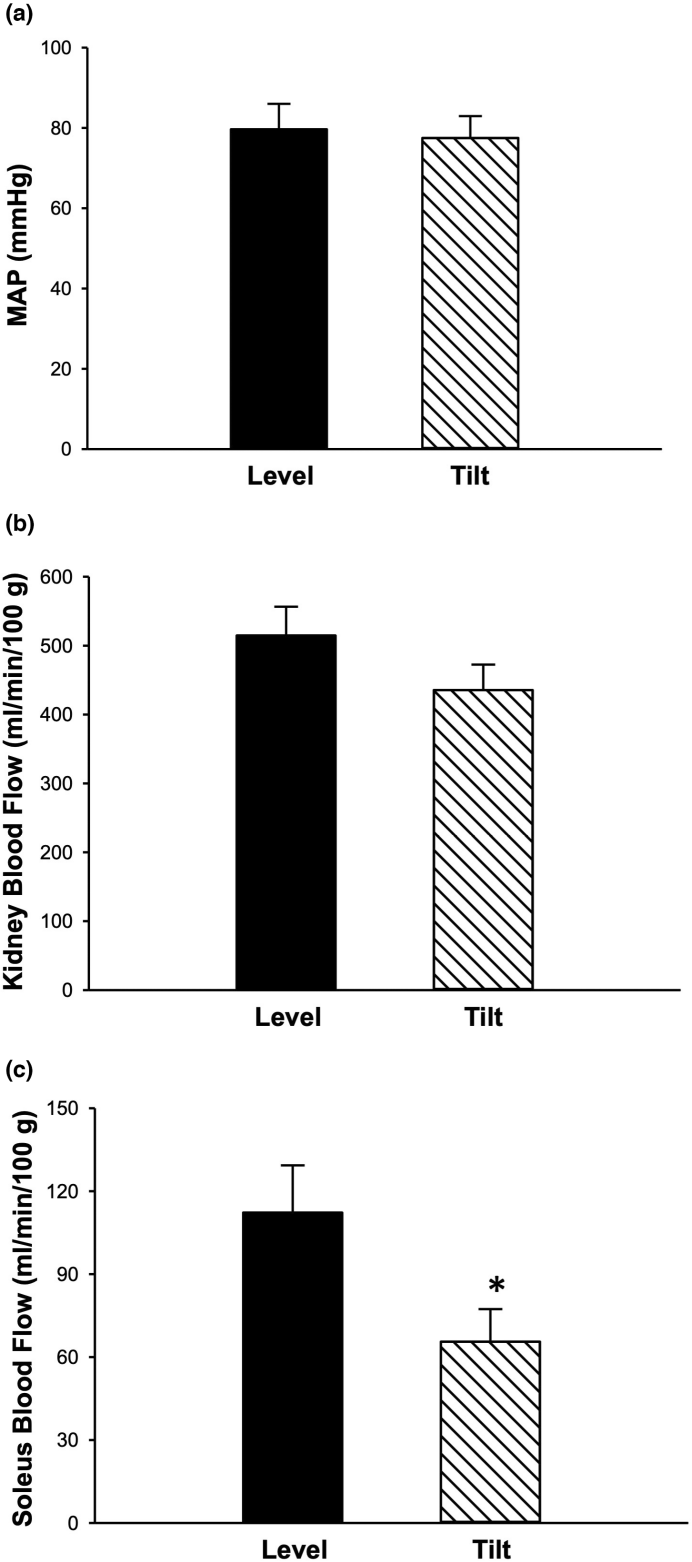
Mean arterial pressure, kidney blood flow, and soleus blood flow. Mean arterial pressure (MAP; a) was not significantly different between the level and HUT position (*n* = 6; *p* > 0.05). Average blood flow of the kidneys (b) and soleus (c) in the level versus HUT position (*n* = 6; *p ≤ 0.05 vs level). Data are mean ± SE.

Heart rate was not different for level versus HUT (426 ± 24 vs 416 ± 26 bpm; *p* = 0.08). There was no change in MAP between level and HUT (80 ± 15 vs 78 ± 13 mmHg; *p* = 0.27; Figure 1a). Average kidney blood flow was not different between body positions (Figure 1b). Soleus blood flow decreased significantly from the level to HUT position (*p* = 0.018; Figure 1c).

#### 3.2.2 Heart to prostate distance, estimated prostate tumor arterial pressure, and prostate tumor perfusion, vascular conductance, and vascular resistance

**FIGURE 2:**
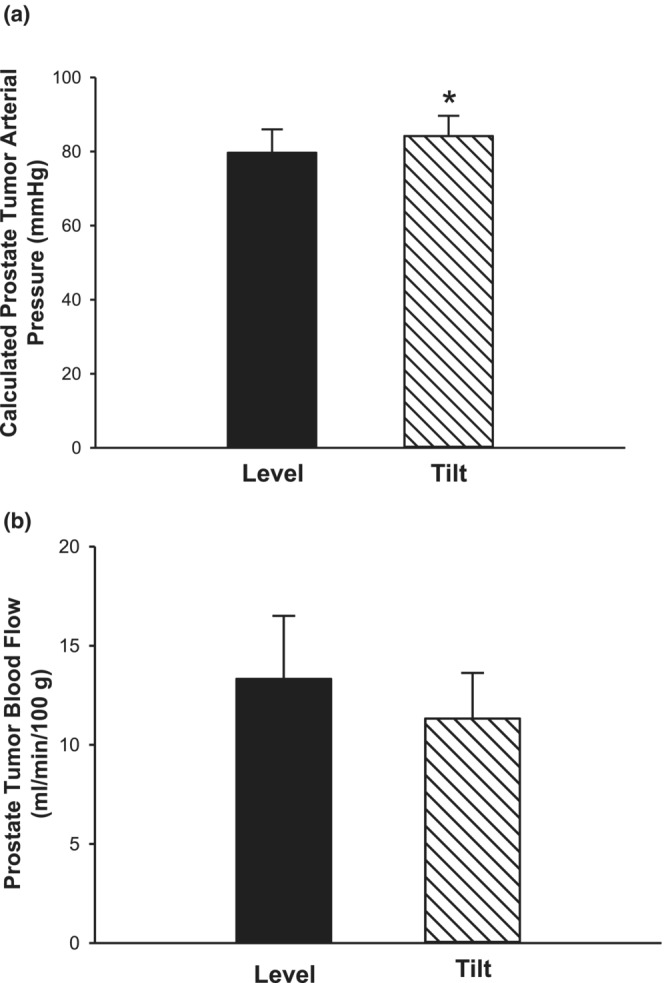
Calculated prostate tumor arterial pressure and perfusion. Calculated arterial pressure (a) and perfusion (b) of the prostate tumor in the level versus HUT position (*n* = 6; *p ≤ 0.05 vs level). Data are mean ± SE.

**FIGURE 3:**
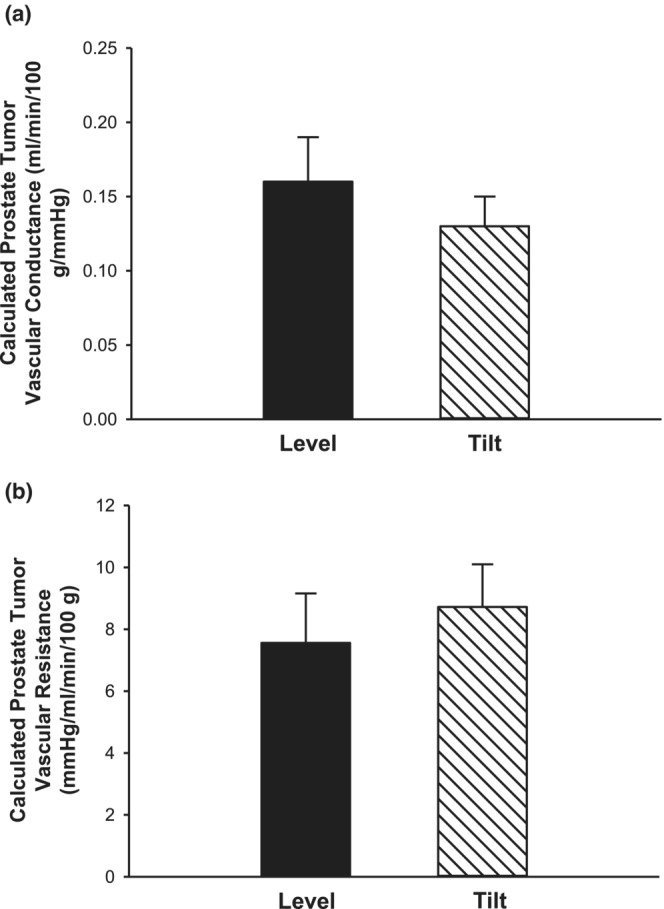
Calculated prostate tumor vascular conductance and resistance. Vascular conductance (a) and vascular resistance (b) of the prostate tumor using calculated tumor arterial pressure were not different in the level versus HUT position (*n* = 6; *p* > 0.05) Data are mean ± SE.

The average distance between the carotid artery cannula tip (where MAP was measured) to the prostate tumor was 9.1 ± 0.8 cm. In the level position, prostate tumor arterial pressure and MAP would be almost identical. However, in the HUT position, the hydrostatic gradient would result in a prostate tumor pressure above that of the MAP. In the HUT position, we report prostate tumor vascular conductance and resistance from the estimated tumor arterial pressure. At 90°, assuming an increase of 2 mmHg per 2.54 cm of vertical distance between the cannula tip and prostate tumor, pressure would increase by an average of 7.2 mmHg. At 70°, the vertical distance is reduced to 8.6 ± 0.7 cm, resulting in increase in tumor arterial pressure of 6.7 ± 0.6 mmHg above measured MAP. Thus, despite no change in MAP between level and HUT (Figure 1a), estimated tumor arterial pressure would be increased in the HUT versus level position (Figure 2a). However, average prostate tumor perfusion did not differ between body positions (Figure 2b). Although there was a slight increase in estimated tumor arterial pressure from the level to HUT position, there were no differences in prostate tumor vascular conductance (Figure 3a) or prostate tumor vascular resistance (Figure 3b) in response to HUT.

To contrast the pressure difference to HUT in rats versus humans, we measured the anatomical distance between the heart and prostate of the animals in this study as well as in humans. The average heart to prostate distance in humans was 48.3 ± 1.2 cm, and the vertical component with 70° HUT was 45.3 ± 1.1 cm. This would result in a hydrostatic pressure difference between the heart and prostate in humans of 35.7 ± 0.9 mmHg in the HUT position. Subject characteristics are summarized in Table 2.

**TABLE 2:** Heart to prostate distance measures

	Human	Rat
*n*	8	6
Height (cm)	178 ± 3.1	
Weight (kg)	83 ± 5.0	0.27 ± 0.01
Anatomical heart to prostate distance (cm)	48 ± 1.2	9.1 ± 0.8
Vertical distance at 70° HUT (cm)	45 ± 1.1	8.6 ± 0.7
Hydrostatic column (mm Hg)	35 ± 0.9	6.7 ± 0.6

*Note*: Data presented as mean ± SEM. A summary of subject characteristics and average heart to prostate distance and pressure gradients.

### 3.3 Tumor hypoxia with HUT

**FIGURE 4:**
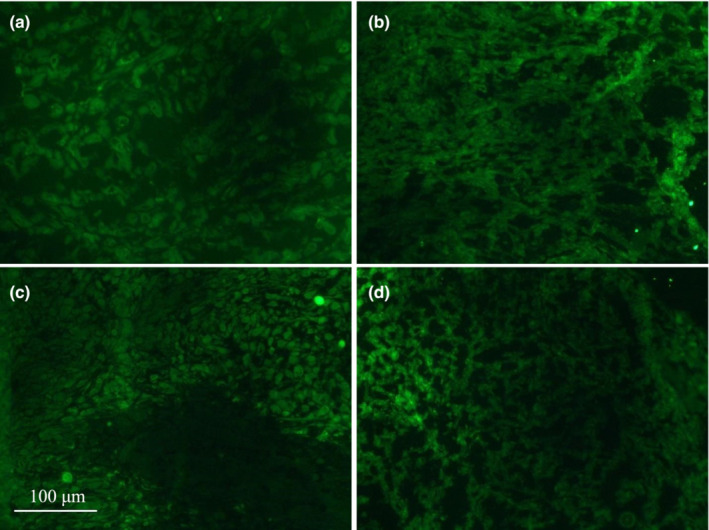
Representative images of hypoxia staining. Representative images for hypoxia staining are shown for core in the level (a) and HUT (b) positions and for periphery in the level (c) and HUT (d) positions. Images were taken at 20× zoom under a fluorescent microscope with a filtered lens.

**FIGURE 5:**
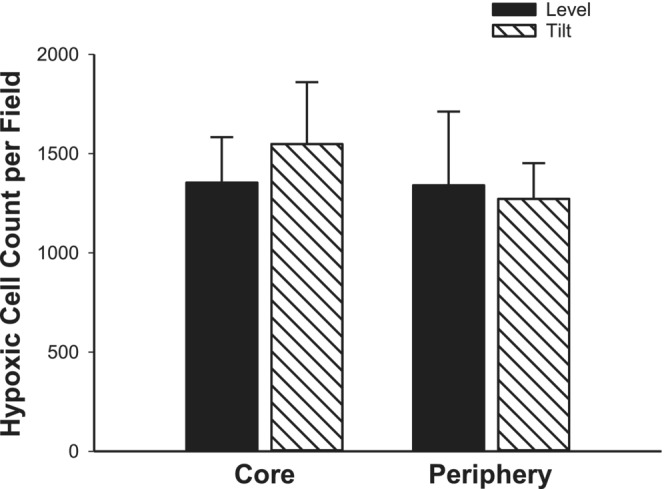
Hypoxic cell count. Hypoxic cell count was not different in core or periphery sections of the prostate tumor in the level (*n* = 4) versus HUT (*n* = 7) positions (*p* > 0.05) Data are mean ± SE.

Figure 4 is a representative image of fluorescent markers of tumor hypoxia assessed using Hypoxyprobe‐1, within the tumor core in level and HUT positions (Figures 4a,b, respectively) and within the tumor periphery in level and HUT positions (Figure 4c,d, respectively). There was no significant difference in hypoxia between core and periphery in the level position (1354 ± 229 vs 1340 ± 372 hypoxic cell counts per field; *p* = 0.99) or in the HUT position (1548 ± 312 vs 1272 ± 180 hypoxic cell counts per field; *p* = 0.97). There was no significant difference in hypoxia between level and HUT in either the core (level, 1354 ± 229 vs HUT, 1548 ± 312 hypoxic cell counts per field; *p* = 0.99) or periphery of tumors (level, 1340 ± 372 vs HUT, 1272 ± 180 hypoxic cell counts per field; *p* = 0.97; Figure 5). There was a large variability in hypoxic count in some groups, but no outliers were detected via analysis using a Grubbs test.

### 3.4 Prostate vascular resistance in young versus aged animals

**FIGURE 6:**
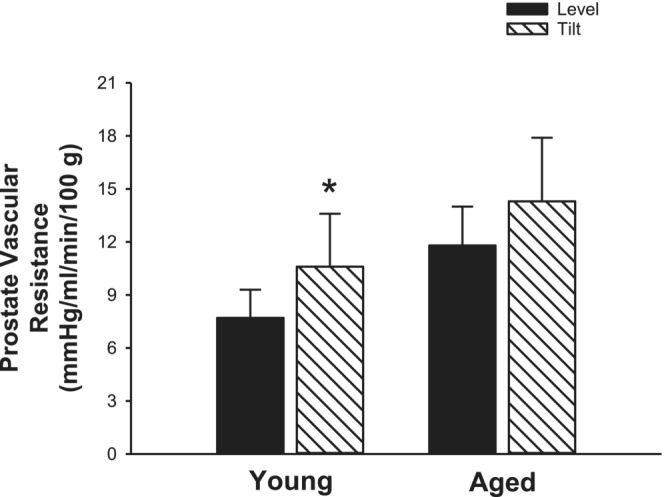
Prostate vascular resistance. Prostate vascular resistance, using estimated prostate arterial pressure, increased in response to HUT in young animals (*n* = 9), but not in aged animals (*n* = 7) (*p ≤ 0.05 vs level within age group). Data are mean ± SE, previously unreported from Ramsey et al. (2007).

There was a significant increase in vascular resistance of the prostate in response to HUT in the young animals (level, 7.7 ± 1.6 vs HUT, 10.6 ± 3.0 mmHg/mL/min/100 g; *p* = 0.048), but not in the aged animals (level, 11.8 ± 2.2 vs HUT, 14.3 ± 3.6 mmHg/mL/min/100 g; p = 0.47) (Figure 6).

## 4 DISCUSSION

### 4.1 Augmenting hydrostatic pressure through posture

To the best of our knowledge, this is the first study to investigate the effects of manipulations in hydrostatic pressure, via 70° HUT, on prostate tumor perfusion and hypoxia. Our investigation revealed that 70° HUT results in a slight increase in estimated prostate tumor arterial pressure, but does not elicit a change in prostate tumor vascular resistance or conductance, nor increase prostate tumor perfusion or decrease prostate tumor hypoxia in young animals.

Moderate‐intensity exercise has been utilized to take advantage of tumor vessel dysfunction, and both acute and chronic exercise have been shown to result in a reduction in tumor hypoxia (McCullough et al., 2013, 2014). Acutely, an increase in cardiac output and MAP with exercise, coupled with dysfunctional tumor arteriolar vasoconstriction, results in a significant increase in prostate tumor perfusion compared to the resting condition.

In the supine position in humans, perfusion pressures are similar across vascular beds. That is, the arterial pressure at any point in the body is nearly equal to the MAP. In the upright position, however, there exists a significant hydrostatic gradient wherein the perfusion pressure of organs and tissues below the level of the heart is higher than those above the heart. Thus, with HUT, the perfusion pressure of tissues/organs below the heart would also increase. Due to the anatomical location of the prostate below the heart, in health, prostate vascular resistance would increase in response to a hemodynamic challenge such as HUT to maintain blood flow at the higher perfusion pressure. However, we hypothesized that in prostate cancer, in response to HUT, there would be an inability to increase vascular resistance thus increasing tumor perfusion. Although we did not observe this in the younger Copenhagen rats, it is likely that the old age‐related changes in prostate vascular function, as inferred from the lack of vasoconstriction to HUT (Figure 6), may lead to an enhanced tumor perfusion in older subjects, and warrants further investigation.

### 4.2 Tumor vascular responses to increased intraluminal pressure

There is a diminished myogenic vasoconstriction in prostate tumor arterioles above intraluminal pressures of 120 cmH_2_O (McCullough et al., 2014). As such, it is unlikely that the increased tumor arterial pressure with 70° HUT of 84.2 mmHg (or 114.5 cmH_2_O), lower than the previously reported value where diminished myogenic tone manifests (McCullough et al., 2014), was enough to overcome the vascular restraints within the tumor resulting, in part, from high intra‐tumoral interstitial fluid pressures. However, the absolute hydrostatic pressure difference at 70° HUT is more than 5× greater in humans than in rats (Table 2). Given rats and humans have similar arterial pressures, a 5x greater hydrostatic gradient in humans would likely result in a tumor perfusion pressure that may enhance tumor perfusion and oxygenation.

### 4.3 Effects of aging

Given the majority of patients with prostate cancer are >60 years of age (Siegel et al., 2018) and aging is associated with both generalized vascular dysfunction (for review see (El Assar et al., 2012)) as well as diminished prostate autonomic innervation (Chapple et al., 1991) and α_1_ adrenoreceptor density (Yono et al., 2006), it is likely that old age may result in disparate prostate arteriolar responses versus younger counterparts. The retrospective analysis of data originally collected by Ramsey et al. (2007) revealed that there was a significant increase in the estimated vascular resistance of the prostate in response to HUT in the young, but not in the aged animals (Figure 6), suggesting a diminished vasoconstriction within the prostate of aged animals. These findings reveal that the ability to maintain healthy prostate vascular resistance in response to 70° HUT is present in young, but not aged rats, consistent with age‐related changes in prostate innervation and smooth muscle contraction (White et al., 2013). As such, age is an important factor to consider in the interpretation of our original findings, as the data from Figure 6 suggest old age‐related prostate vascular dysfunction that would likely not be present in the younger Copenhagen rats used in this study. Specifically, the age of the young, immunocompetent Copenhagen rats used in the initial study would be similar to a young adult human, which is considerably younger than the average prostate cancer patient with an average age of 66 years (Rawla, 2019). As tumors co‐opt existing functional mature vessels as well as form de novo dysfunctional vessels (Sonveaux, 2008), it is possible that a lack of old age‐related prostate vascular dysfunction in the Copenhagen rats contributed to the absence of any changes in tumor perfusion. Although there is not a well‐established animal model to study the combined effects of aging and prostate cancer, the secondary analysis revealed a clear difference in the hemodynamic response of the *healthy* prostate to HUT in young versus aged animals. Specifically, in response to HUT, healthy prostate vascular resistance increased in the young animals, and did not change in the old animals, indicating potential vascular dysfunction of the prostate that occurs with aging. Though our study did not reveal a significant increase in tumor vascular resistance in response to HUT, the combined effect of age and dysfunctional tumor vasculature may be a significant contributing factor to the response to HUT in animals (and humans) with prostate cancer.

## 5 LIMITATIONS

There are several limitations in this study. Though the findings from this study suggest that the combination of aged and tumor‐bearing rats may reveal an increased prostate tumor perfusion in response to HUT that was not seen in this study, we did not perform additional studies using aged tumor‐bearing rats as, currently, there are no aged models of prostate cancer in immunocompetent rats. Secondly, there is a smaller sample size for tumor hypoxia in the level (*n* = 4) versus HUT position (*n* = 7), as well as a small sample size for the perfusion experiments; unfortunately, during the collection of the hypoxia data, the Copenhagen rat strain was discontinued and was no longer available to increase sample size. However, the absence of change in blood flow to the prostate tumor between level and HUT positions (Figure 2) suggests no change in gross tumor oxygen delivery, which supports the lack of change in tumor hypoxia with HUT (Figure 5). Additionally, the prostate tumor arterial pressure is estimated and minimal, and the sample size is small. A larger stimulus may be requisite to reach pressures above which myogenic vasoconstriction is diminished in the prostate tumor.

Animals subjected to blood flow measurements were given acepromazine in order to reduce stress/anxiety during the tilt protocol and minimize the risk of catheter displacement due to excessive movement. The antagonistic effect of acepromazine on α_1_ receptors may have contributed to an inability to augment vascular resistance in some non‐vital tissues in response to HUT. Additionally, the administration of acepromazine prior to any measurements resulted in a reduction in MAP, which was below the MAP normally requisite to reveal any dysfunction in myogenic regulation.

## 6 CONCLUSIONS

Although this study revealed that 70° HUT did not augment the vascular hydrostatic pressure gradient or increase prostate tumor perfusion or decrease prostate tumor hypoxia, the overall change in the estimated hydrostatic pressure difference was minimal. This suggests that the hemodynamic stimulus imparted by 70° HUT may not be requisite to elicit alterations in prostate tumor vascular function, and thus, tumor perfusion. In addition, the secondary analysis performed in non‐tumor‐bearing animals revealed that the age of the animals may be a key component in the ability to augment vascular resistance in response to HUT. Tumors in aged animals would both co‐opt vessels from the aged host tissue, as well as form new dysfunctional vessels via hypoxia‐mediated signaling. The resultant tumor vasculature may therefore be characterized by a level of dysfunction conducive to augmenting tumor perfusion in response to HUT. Additionally, given that humans would experience a much larger prostate tumor arterial pressure than rats, HUT may still be a valuable tool to increase prostate tumor perfusion and decrease tumor hypoxia in humans, both of which are important for treatment efficacy and patient prognosis. Further studies should be performed in an aged model of prostate cancer to determine whether HUT might augment the vascular hydrostatic pressure gradient in older patients.

## AUTHOR CONTRIBUTIONS

ONK, TAR, JGP, MWR, MDD, and BJB conceived and designed the research. ONK, TAR, JGP, DRB, AOA, CJA, TIM, and BJB performed experiments. ONK, TAR, JGP, TIM, and BJB interpreted experimental results. ONK, TAR, and JGP prepared figures and drafted manuscript. ONK, TAR, JGP, DRB, AGH, AOA, CJA, TIM. MWR, MDD, and BJB edited and revised manuscript. ONK, TAR, JGP, DRB, AGH, AOA, CJA, TIM. MWR, MDD, and BJB approved the final version of manuscript.

## FUNDING INFORMATION

This work was supported by the National Institutes of Health (R15 AG 078060‐A1), the American Cancer Society (RSG‐14‐150‐01‐CCE), and the Johnson Cancer Research Center at Kansas State University.

## CONFLICT OF INTEREST

We report no disclosures or conflict of interest.

## ETHICAL STATEMENT

All animal procedures were performed in accordance with Institutional Animal Care and Use Committee guidelines. Informed consent was obtained for all human subjects and all procedures were performed in accordance with the ethical standards of the responsible committee on human experimentation and with the Helsinki Declaration of 1975, as revised in 2008.

## References

[phy215548-bib-0001] Baish, J. W. , Gazit, Y. , Berk, D. A. , Nozue, M. , Baxter, L. T. , & Jain, R. K. (1996). Role of tumor vascular architecture in nutrient and drug delivery: An invasion percolation‐based network model. Microvascular Research, 51(3), 327–346. 10.1006/mvre.1996.0031 8992232

[phy215548-bib-0002] Baluk, P. , Morikawa, S. , Haskell, A. , Mancuso, M. , & McDonald, D. M. (2003). Abnormalities of basement membrane on blood vessels and endothelial sprouts in tumors. The American Journal of Pathology, 163(5), 1801–1815. 10.1016/s0002-9440(10)63540-7 14578181 PMC1892429

[phy215548-bib-0003] Bristow, R. G. , & Hill, R. P. (2008). Hypoxia and metabolism. Hypoxia, DNA repair and genetic instability. Nature Reviews Cancer, 8(3), 180–192. 10.1038/nrc2344 18273037

[phy215548-bib-0004] Carmeliet, P. , & Jain, R. K. (2000). Angiogenesis in cancer and other diseases. Nature, 407(6801), 249–257. 10.1038/35025220 11001068

[phy215548-bib-0005] Chapple, C. R. , Crowe, R. , Gilpin, S. A. , Gosling, J. , & Burnstock, G. (1991). The innervation of the human prostate gland—The changes associated with benign enlargement. The Journal of Urology, 146(6), 1637–1644. 10.1016/s0022-5347(17)38203-4 1719253

[phy215548-bib-0006] Deveci, D. , & Egginton, S. (1999). Development of the fluorescent microsphere technique for quantifying regional blood flow in small mammals. Experimental Physiology, 84(4), 615–630. 10481220

[phy215548-bib-0007] El Assar, M. , Angulo, J. , Vallejo, S. , Peiró, C. , Sánchez‐Ferrer, C. F. , & Rodríguez‐Mañas, L. (2012). Mechanisms involved in the aging‐induced vascular dysfunction. Frontiers in Physiology, 3, 132. 10.3389/fphys.2012.00132 22783194 PMC3361078

[phy215548-bib-0008] Höckel, M. , & Vaupel, P. (2001). Tumor hypoxia: Definitions and current clinical, biologic, and molecular aspects. Journal of the National Cancer Institute, 93(4), 266–276. 10.1093/jnci/93.4.266 11181773

[phy215548-bib-0009] Isaacs, J. T. , Heston, W. D. , Weissman, R. M. , & Coffey, D. S. (1978). Animal models of the hormone‐sensitive and ‐insensitive prostatic adenocarcinomas, dunning R‐3327‐H, R‐3327‐HI, and R‐3327‐AT. Cancer Research, 38(11 Pt 2), 4353–4359. 698976

[phy215548-bib-0010] Jain, R. K. (1988). Determinants of tumor blood flow: A review. Cancer Research, 48(10), 2641–2658. 3282647

[phy215548-bib-0011] Jansen, R. W. , Lenders, J. W. , Thien, T. , & Hoefnagels, W. H. (1989). The influence of age and blood pressure on the hemodynamic and humoral response to head‐up tilt. Journal of the American Geriatrics Society, 37(6), 528–532. 10.1111/j.1532-5415.1989.tb05684.x 2715560

[phy215548-bib-0012] Kalbfleisch, J. H. , Reinke, J. A. , Porth, C. J. , Ebert, T. J. , & Smith, J. J. (1977). Effect of age on circulatory response to postural and valsalva tests (39884). Proceedings of the Society for Experimental Biology and medicine Society for Experimental Biology and Medicine (New York, NY), 156(1), 100–103. 10.3181/00379727-156-39884 909876

[phy215548-bib-0013] Konerding, M. A. , Malkusch, W. , Klapthor, B. , van Ackern, C. , Fait, E. , Hill, S. A. , et al. (1999). Evidence for characteristic vascular patterns in solid tumours: Quantitative studies using corrosion casts. British Journal of Cancer, 80(5–6), 724–732. 10.1038/sj.bjc.6690416 10360650 PMC2362271

[phy215548-bib-0014] Li, C. Y. , Shan, S. , Huang, Q. , Braun, R. D. , Lanzen, J. , Hu, K. , Lin, P. , & Dewhirst, M. W. (2000). Initial stages of tumor cell‐induced angiogenesis: Evaluation via skin window chambers in rodent models. Journal of the National Cancer Institute., 92(2), 143–147. 10.1093/jnci/92.2.143 10639516

[phy215548-bib-0015] Lipsitz, L. A. (1985). Abnormalities in blood pressure homeostasis that contribute to falls in the elderly. Clinics in Geriatric Medicine, 1(3), 637–648. 3913513

[phy215548-bib-0016] Ljungkvist, A. S. , Bussink, J. , Kaanders, J. H. , Rijken, P. F. , Begg, A. C. , Raleigh, J. A. , et al. (2005). Hypoxic cell turnover in different solid tumor lines. International Journal of Radiation Oncology, Biology, Physics, 62(4), 1157–1168. 15913908 10.1016/j.ijrobp.2005.03.049

[phy215548-bib-0017] Martel, E. , Ponchon, P. , Champéroux, P. , Elghozi, J. L. , de la Faverie JF, R. , Dabiré, H. , Pannier, B. , Richard, S. , Safar, M. , & Cuche, J. L. (1998). Mechanisms of the cardiovascular deconditioning induced by tail suspension in the rat. The American Journal of Shysiology, 274(5), H1667–H1673. 10.1152/ajpheart.1998.274.5.H1667 9612378

[phy215548-bib-0018] McCullough, D. J. , Nguyen, L. M. , Siemann, D. W. , & Behnke, B. J. (2013). Effects of exercise training on tumor hypoxia and vascular function in the rodent preclinical orthotopic prostate cancer model. Journal of Applied Physiology, 115(12), 1846–1854. 10.1152/japplphysiol.00949.2013 24177690 PMC3882937

[phy215548-bib-0019] McCullough, D. J. , Stabley, J. N. , Siemann, D. W. , & Behnke, B. J. (2014). Modulation of blood flow, hypoxia, and vascular function in orthotopic prostate tumors during exercise. Journal of the National Cancer Institute, 106(4), dju036. 10.1093/jnci/dju036 24627275 PMC3982888

[phy215548-bib-0020] Minson, C. T. , Wladkowski, S. L. , Pawelczyk, J. A. , & Kenney, W. L. (1999). Age, splanchnic vasoconstriction, and heat stress during tilting. The American Journal of Physiology, 276(1 Pt 2), R203–R212. 10.1152/ajpregu.1999.276.1.r203 9887196

[phy215548-bib-0021] Musacchia, X. J. , Steffen, J. M. , & Dombrowski, J. (1992). Rat cardiovascular responses to whole body suspension: Head‐down and non‐head‐down tilt. Journal of Applied Physiology, 73(4), 1504–1509. 10.1152/jappl.1992.73.4.1504 1447098

[phy215548-bib-0022] Ramsey, M. W. , Behnke, B. J. , Prisby, R. D. , & Delp, M. D. (2007). Effects of aging on adipose resistance artery vasoconstriction: Possible implications for orthostatic blood pressure regulation. Journal of Applied Physiology, 103(5), 1636–1643. 10.1152/japplphysiol.00637.2007 17885023

[phy215548-bib-0023] Rawla, P. (2019). Epidemiology of prostate cancer. World Journal of Oncology, 10(2), 63–89. 10.14740/wjon1191 31068988 PMC6497009

[phy215548-bib-0024] Rowell, L. B. (1993). Human cardiovascular control. Oxford University Press.

[phy215548-bib-0025] Semenza, G. L. (2003). Targeting HIF‐1 for cancer therapy. Nature Reviews Cancer, 3(10), 721–732. 10.1038/nrc1187 13130303

[phy215548-bib-0026] Siegel, R. L. , Miller, K. D. , & Jemal, A. (2018). Cancer statistics, 2018. CA: A Cancer Journal for Clinicians, 68(1), 7–30. 10.3322/caac.21442 29313949

[phy215548-bib-0027] Siemann, D. W. (2011). The unique characteristics of tumor vasculature and preclinical evidence for its selective disruption by tumor‐vascular disrupting agents. Cancer Treatment Reviews, 37(1), 63–74. 10.1016/j.ctrv.2010.05.001 20570444 PMC2958232

[phy215548-bib-0028] Sonveaux, P. (2008). Provascular strategy: Targeting functional adaptations of mature blood vessels in tumors to selectively influence the tumor vascular reactivity and improve cancer treatment. Radiotherapy and Oncology, 86(3), 300–313. 10.1016/j.radonc.2008.01.024 18313779

[phy215548-bib-0029] Thomlinson, R. H. , & Gray, L. H. (1955). The histological structure of some human lung cancers and the possible implications for radiotherapy. British Journal of Cancer, 9(4), 539–549. 10.1038/bjc.1955.55 13304213 PMC2073776

[phy215548-bib-0030] Turaka, A. , Buyyounouski, M. K. , Hanlon, A. L. , Horwitz, E. M. , Greenberg, R. E. , & Movsas, B. (2012). Hypoxic prostate/muscle PO_2_ ratio predicts for outcome in patients with localized prostate cancer: Long‐term results. International Journal of Radiation Oncology, Biology, Physics, 82(3), e433–e439. 10.1016/j.ijrobp.2011.05.037 21985947

[phy215548-bib-0031] Vaupel, P. (2004a). Tumor microenvironmental physiology and its implications for radiation oncology. Seminars in Radiation Oncology, 14(3), 198–206. 10.1016/j.semradonc.2004.04.008 15254862

[phy215548-bib-0032] Vaupel, P. (2004b). The role of hypoxia‐induced factors in tumor progression. The Oncologist, 9(Suppl 5), 10–17. 10.1634/theoncologist.9-90005-10 15591418

[phy215548-bib-0033] Vaupel, P. , & Harrison, L. (2004). Tumor hypoxia: Causative factors, compensatory mechanisms, and cellular response. The Oncologist, 9(Suppl 5), 4–9. 10.1634/theoncologist.9-90005-4 15591417

[phy215548-bib-0034] Vaupel, P. , Mayer, A. , & Höckel, M. (2004). Tumor hypoxia and malignant progression. Methods in Enzymology, 381, 335–354. 10.1016/s0076-6879(04)81023-1 15063685

[phy215548-bib-0035] Walsh, J. C. , Lebedev, A. , Aten, E. , Madsen, K. , Marciano, L. , & Kolb, H. C. (2014). The clinical importance of assessing tumor hypoxia: Relationship of tumor hypoxia to prognosis and therapeutic opportunities. Antioxidants & Redox Signaling, 21(10), 1516–1554. 10.1089/ars.2013.5378 24512032 PMC4159937

[phy215548-bib-0036] Watenpaugh, D. E. , & Hargens, A. R. (1996). The cardiovascular system in microgravity. In Handbook of physiology. Environmental physiology (Sect. 4, Vol. I, Chapt. 29, pp. 631–674). American Physiological Society.

[phy215548-bib-0037] White, C. W. , Xie, J. H. , & Ventura, S. (2013). Age‐related changes in the innervation of the prostate gland: Implications for prostate cancer initiation and progression. Organogenesis, 9(3), 206–215. 10.4161/org.24843 23872639 PMC3896592

[phy215548-bib-0038] Wilkerson, M. K. , Muller‐Delp, J. , Colleran, P. N. , & Delp, M. D. (1999). Effects of hindlimb unloading on rat cerebral, splenic, and mesenteric resistance artery morphology. Journal of Applied Physiology, 87(6), 2115–2121. 10.1152/jappl.1999.87.6.2115 10601157

[phy215548-bib-0039] Yancopoulos, G. D. , Davis, S. , Gale, N. W. , Rudge, J. S. , Wiegand, S. J. , & Holash, J. (2000). Vascular‐specific growth factors and blood vessel formation. Nature, 407(6801), 242–248. 10.1038/35025215 11001067

[phy215548-bib-0040] Yono, M. , Foster, H. E., Jr. , Weiss, R. M. , & Latifpour, J. (2006). Age related changes in the functional, biochemical and molecular properties of alpha1‐adrenoceptors in the rat genitourinary tract. The Journal of Urology, 176(3), 1214–1219. 10.1016/j.juro.2006.04.038 16890728

